# Supplementary material for: Empirical mean-noise fitness landscapes reveal the fitness impact of gene expression noise
Source: Nat Commun. 2019 Jul 18;10:3180. doi: 10.1038/s41467-019-11116-w (PMC6639414; doi:10.1038/s41467-019-11116-w)
Supplement: Supplementary file 1 — Supplementary Information [file 41467_2019_11116_MOESM1_ESM.pdf]

Supplementary Information

Empirical mean-noise fitness landscapes reveal the fitness impact of gene expression noise

Schmiedel *et al.*

Table of Contents

Processing of experimental data 2

Quantifying fitness effects of noise in alternative ways 3

Uncertainty of expression-fitness landscapes 4

Expression sensitivity and noise intolerance on expression-fitness landscapes 5

Principal component analysis of expression-fitness landscapes 7

Evolution of gene expression on principal topologies 8

Reversal of noise-fitness effects far away from optimal mean expression 9

Supplementary Table 1 - Data sources 10

Supplementary Note 1 10

Supplementary References 11

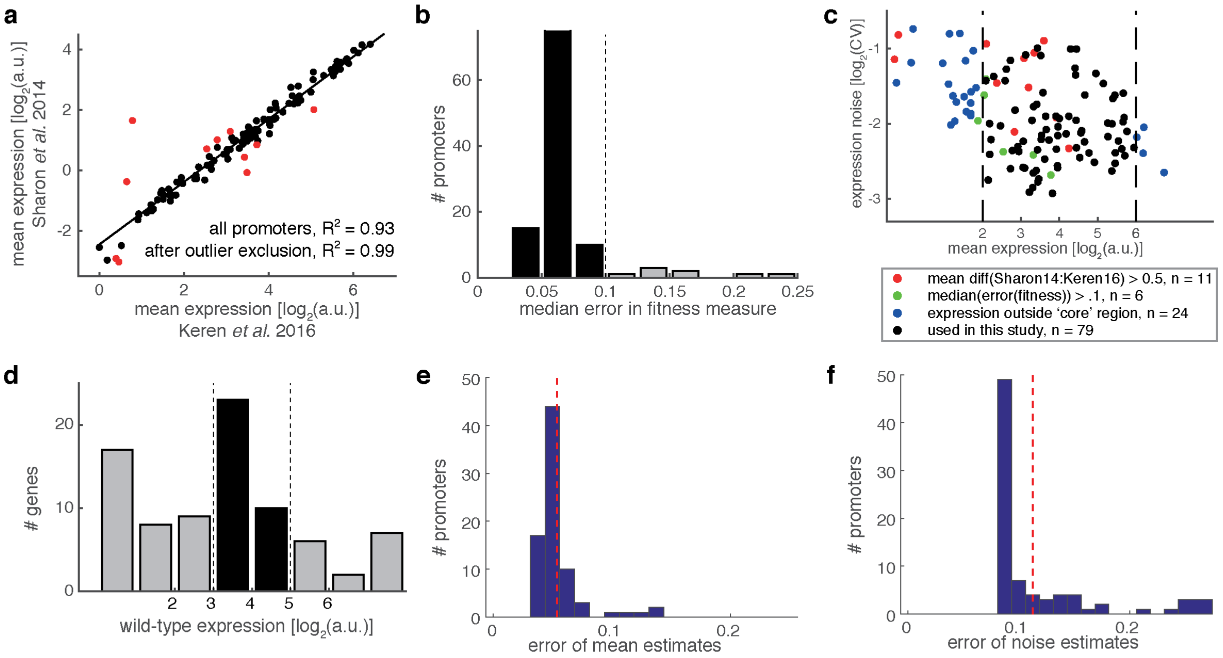

Supplementary Figure 1

Processing of experimental data

a Relative mean expression strength of synthetic promoters when driving a genome-integrated *YFP* measured for individual strains[1] versus when driving a plasmid-based *YFP* measured in a pooled fashion with a fluorescent activated cell sorting and sequencing approach[2]. Outliers excluded from further analysis (because their deviation is greater than 0.5 log_2-units from proportionality relationship) are marked in red. Squared Pearson correlation coefficients are indicated.

b Median error of fitness estimates for each promoter across all 85 genes. Promoters with median errors greater than 0.1 were discarded from analysis.

c Promoters in mean-noise expression space. Promoters excluded because of too large discrepancies in mean expression strengths between the two studies (panel **a**) are marked in red (n = 11). Promoters with too high errors of fitness measurements (panel **b**) are marked in green (n = 6). Promoters that lie outside of the core mean-noise space region analysed (mean range [2,6] log_2-units) are marked in blue (n = 24). The remaining 79 promoters used in this study (black) are homogenously distributed in mean-noise space.

d Estimated wild-type expression strength of endogenous promoters of the 85 genes investigated by Keren, et al. [1]. We focused on genes whose promoters have an expression strength in the centre (mean range [3,5] log_2 units) of the mean-noise space region analysed.

e Error of promoter mean expression values as estimated from running average of replicate standard deviation as a function of sequencing read based error estimate over all promoters. Red bar indicates average error across all promoters: 5.4 %.

f Error of promoter noise values. Average error across all promoters: 11%.

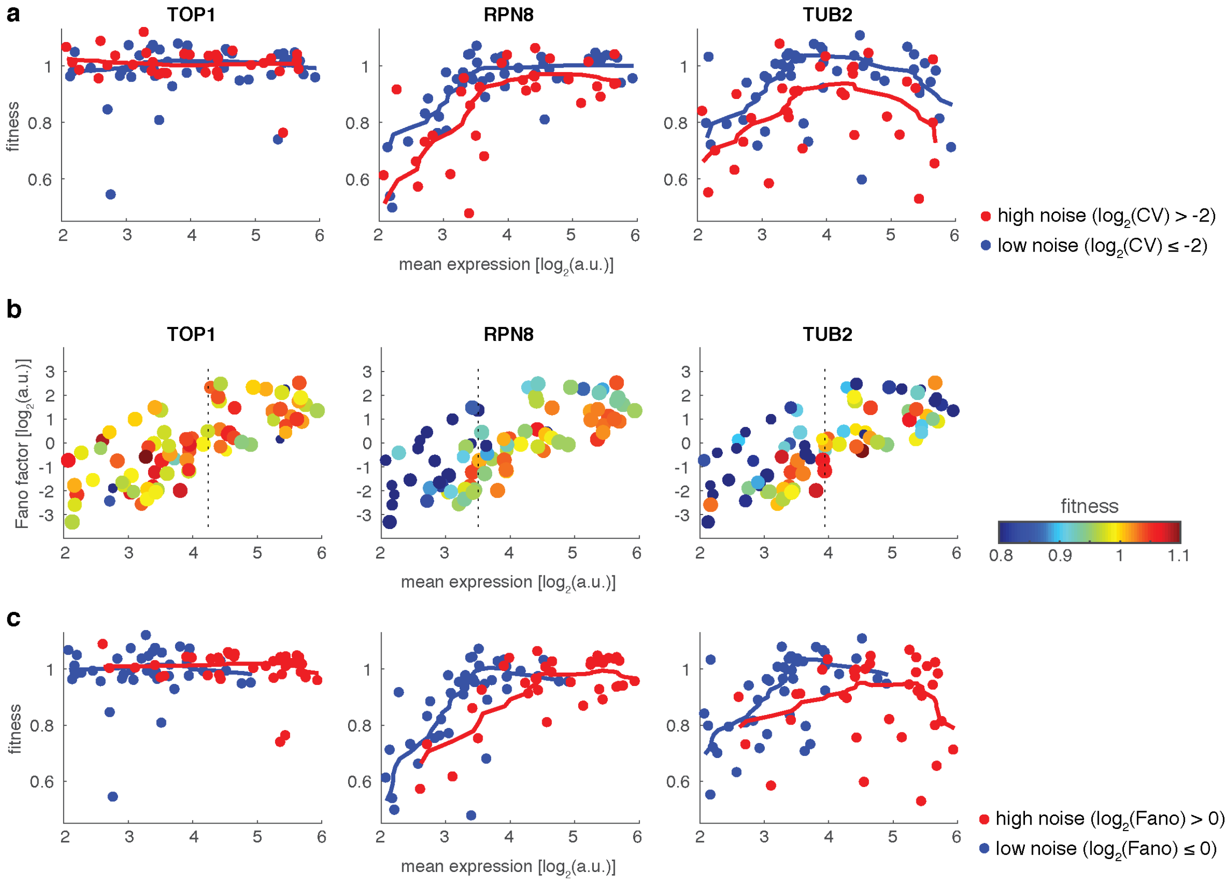

Supplementary Figure 2

Quantifying fitness effects of noise in alternative ways

a Fitness as a function of mean expression when promoters are split according to their noise levels. Dots show individual promoters, line shows a robust loess fit (Matlab function *smooth* with 'span' = 0.9).

b Mean-noise-fitness data with Fano factor (variance divided by mean) as noise metric. Note that Fano factor is plotted as arbitrary units (a.u.), because molecule numbers were not quantified in absolute terms. Dashed vertical line indicates genes' wild-type mean expression.

c Fitness as a function of mean expression when promoters are split according to Fano factors. Dots show individual promoters, line shows a robust loess fit (Matlab function *smooth* with 'span' = 0.9).

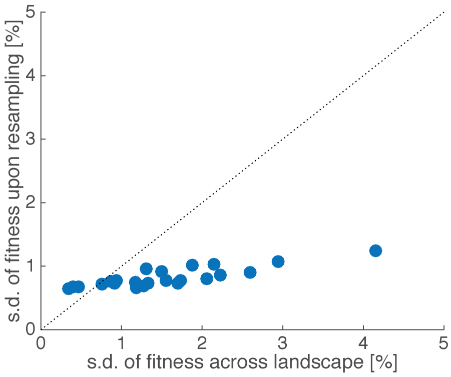

Supplementary Figure 3

Uncertainty of expression-fitness landscapes

Comparison of systematic variation of fitness values across landscapes and the average uncertainty of fitness values on landscapes as estimated from 100 resampled fitness landscapes. Resampling was performed by first drawing with replacement 78 promoters, then, for each promoter-gene strain draw a mean, noise and fitness value from normal distributions according to their experimental estimates and associated errors. Average uncertainty was calculated as standard deviation of fitness values at each grid point over the 100 resampling runs, averaged over all grid points. Systematic variation of fitness values across landscapes (x-axis) is the standard deviation of fitness values at all grid points of a landscape.

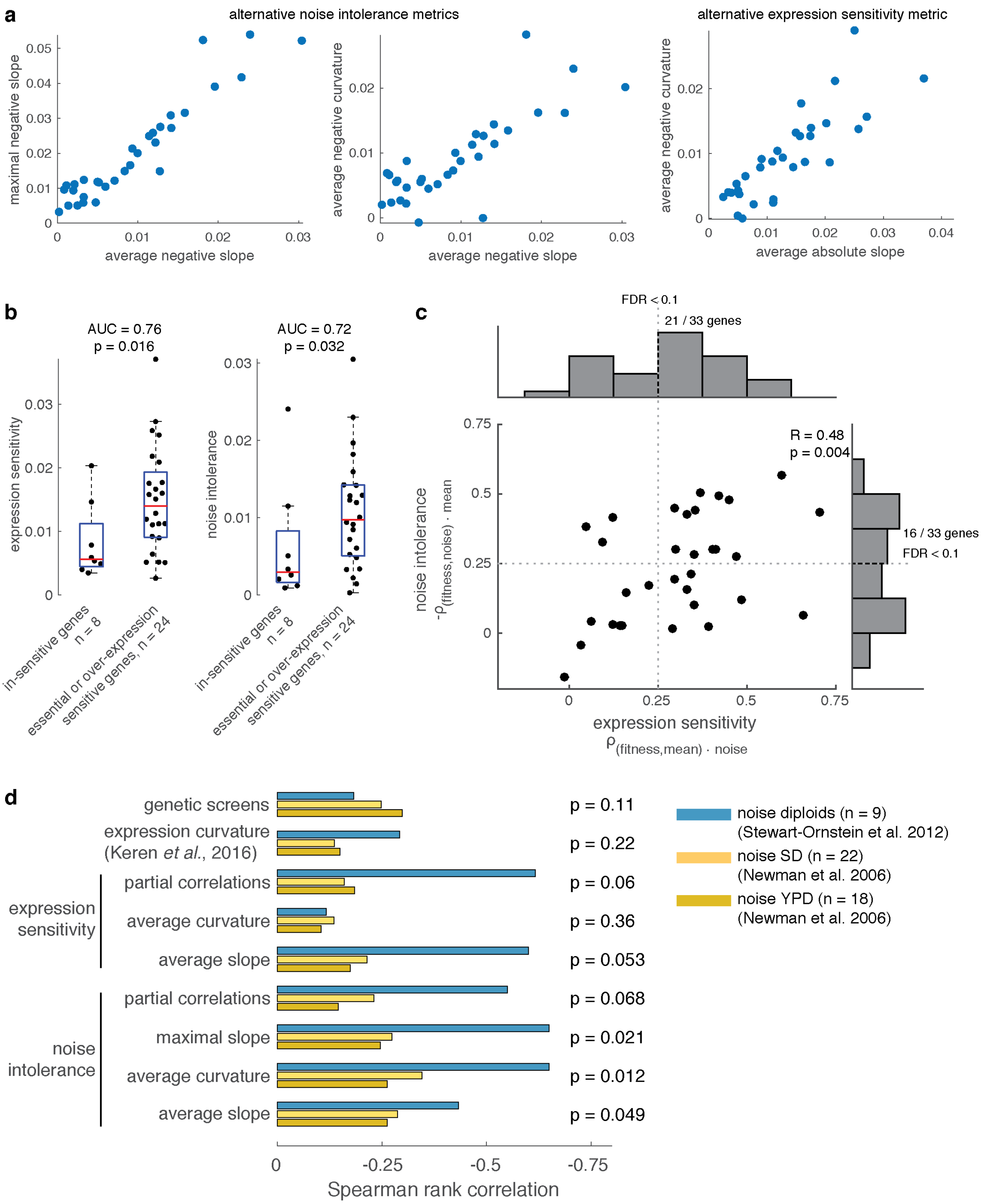

Supplementary Figure 4

Expression sensitivity and noise intolerance on expression-fitness landscapes

a We evaluated several metrics for quantifying the impact of high noise or non-optimal mean expression levels on fitness, which are all correlated and are similarly predictive of endogenous noise levels (see panel **d**). Left, noise intolerance metrics: 'Average negative slope' is the noise intolerance metric described and used in the main text, i.e. we quantified the fitness effect of increasing noise by a factor of two (at wild-type mean expression) by calculating the average decrease (slope) of fitness with increasing mean expression $ni = \left| -{\partial f}/{\partial n} \right|_{m=wt} \right|$.

Similarly, ‘maximum negative slope’ is the maximum of the negative slope of the noise-fitness function at wild-type mean expression $ni = max\left(-\partial f/\partial n\big|_{m=wt}\right)$, thus describing the maximum sensitivity of fitness to noise at any point along the noise-fitness function. ‘Average negative curvature’ describes the concavity of the noise-fitness function, i.e. how much worse fitness sensitivities to noise gets with increasing noise levels, $ni = \left|-\partial^2 f/\partial n^2\big|_{m=wt}\right|$. Right, expression-sensitivity metrics: ‘Average absolute slope’ is the expression-sensitivity metric used in the main text, i.e. the fitness loss when changing mean expression by a factor of two (at low noise levels), no matter which direction, $ni = \left|abs\left(\partial f/\partial m\big|_{n=-3}\right)\right|$. ‘Mean negative curvature’ is the negative of the second derivative of the mean-fitness function at low noise, i.e. $ni = \left|-\partial^2 f/\partial n^2\big|_{m=wt}\right|$, similar to expression-sensitivity metrics used elsewhere[1,3].

b Expression sensitivity and noise intolerance of known dosage sensitive (essential and/or over-expression sensitive) genes. Area under the curve (AUC) of receiver operating characteristic (Matlab function *perfcurve*) as well as one-sided p-value from Wilcoxon rank sum test (Matlab function *ranksum*) are indicated. Boxplots: boxes cover 1st to 3rd quartile of the data, with middle bar indicating median, whiskers extend at maximum to 1.5 times the inter quartile range away from the box.

c Relationship between expression sensitivity and noise intolerance across all genes when derived from Pearson partial correlation of mean or noise with fitness values while controlling for the other expression phenotype from raw gene-promoter strain data. False discovery rate (FDR) < 10% threshold indicated in both dimensions (Matlab function *mafdr* using Benjamini-Hochberg correction). Histograms on top and to the right show distributions of genes across expression sensitivity and noise intolerance, respectively. Indicated are the numbers of genes with FDR < 10%. Pearson correlation coefficient of expression sensitivity and noise intolerance across genes and p-value are indicated.

d Spearman rank correlation between predictors for genes’ intolerance of noise (noise intolerance and expression sensitivity from this study, curvature of fitted expression-fitness functions[1], dosage sensitivity of genes assessed in large-scale genetic screens) and three sets of endogenous noise measurements (n: number of genes with available noise measurements). P-value given is the aggregated p-value from Fisher’s method across all three datasets.

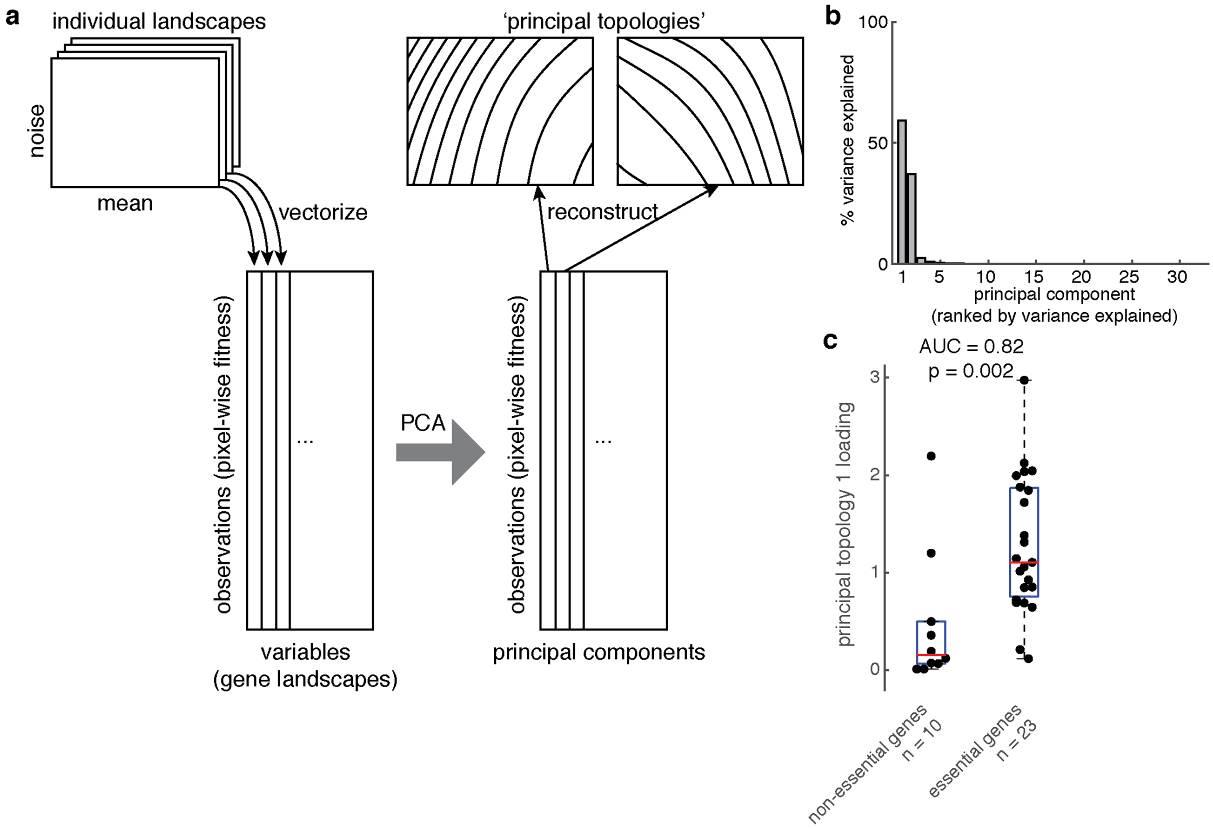

Supplementary Figure 5

Principal component analysis of expression-fitness landscapes

a Overview of principal component analysis. Individual landscapes are vectorised and concatenated into an N x L matrix, with N the number of grid points on the landscapes and L the number of landscapes (33). Principal component analysis on this matrix yields a matrix of principal components, the first two from which the 'principal topologies' are reconstructed.

b Percent variance of fitness landscapes explained by the principal components.

c Principal topology 1 loadings of genes predict genes' essentiality as assessed from gene deletion screens[4]. Area under the curve (AUC) of receiver operating characteristic (Matlab function *perfcurve*) as well as one-sided p-value from Wilcoxon rank sum test (Matlab function *ranksum*) are indicated. Boxplots: boxes cover 1st to 3rd quartile of the data, with middle bar indicating median, whiskers extend at maximum to 1.5 times the inter quartile range away from the box.

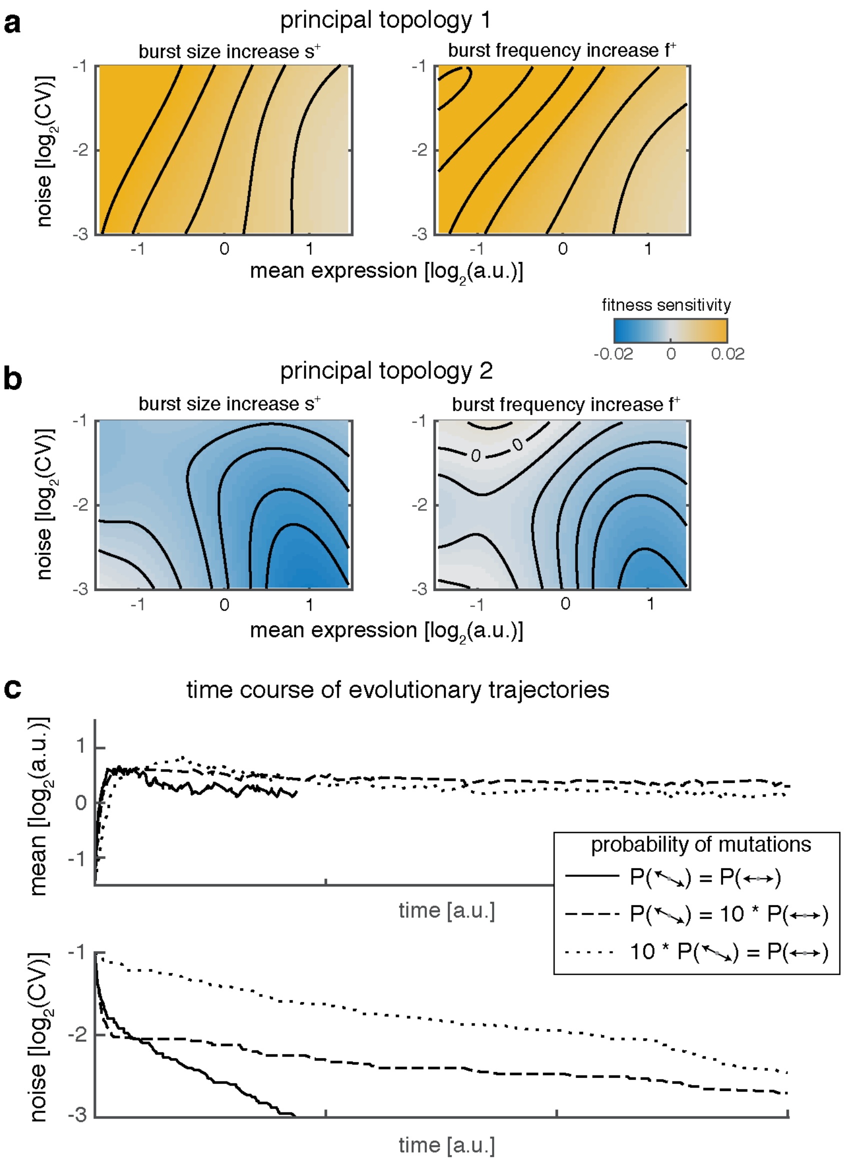

Supplementary Figure 6

Evolution of gene expression on principal topologies

a,b Fitness gains of mutations increasing either burst size (s^+, left) or burst frequency (f^+, right) across the principal topology 1 or 2 landscapes. In contrast to peaked landscapes, no misalignment of regions where burst frequency and burst size altering mutations are beneficial or detrimental are detected.

c Temporal evolution of mean expression and noise on peaked landscape for example trajectories starting at high noise and low fitness (upper left corner in Fig. 6d). Solid line: Equal probability for burst size and burst frequency mutations to occur, as shown in Fig. 6d. Dashed and dotted lines: Burst frequency or burst size mutations ten times more likely to occur, respectively.

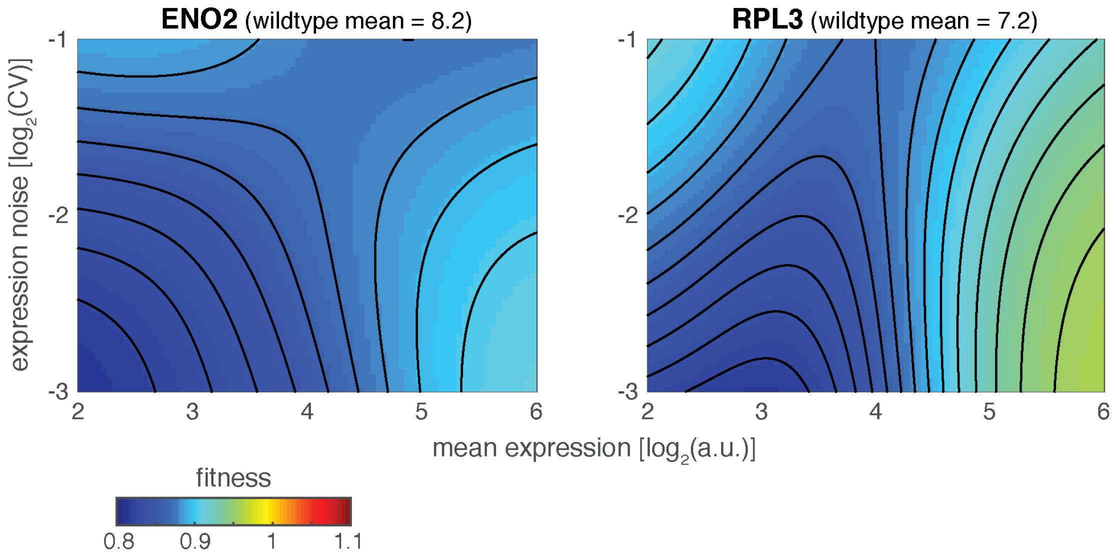

Supplementary Figure 7

Reversal of noise-fitness effects far away from optimal mean expression

Expression-fitness landscapes of two genes (ENO2, wildtype mean expression = 8.2 log_2-units; RPL3 wildtype mean expression = 7.2 log_2-units) for which high noise levels transition from being detrimental close to wild-type mean expression (mean expression above ~5 log_2-units) to beneficial far below wild-type mean expression (mean expression below ~3.5 log_2-units).

Supplementary Table 1 - Data sources

Data	Source
Mean expression levels of endogenous gene promoters and synthetic promoters when driving *YFP* from *HIS3* locus	Keren, et al. [1]
Read counts of gene-promoter combinations in competitive growth experiments on glucose (used to calculate strain fitness)	Gene Expression Omnibus accession number GSE83936 [1]
Mean expression and noise levels of synthetic promoters driving *YFP* from plasmid	Sharon, et al. [2]
Synthetic promoter sequences, list of transcription factor motives	Sharon, et al. [5]
Protein expression noise of GFP-fusions in haploid cells	Newman, et al. [6]
Protein expression noise of GFP-fusions in diploid cells	Stewart-Ornstein, et al. [7]
Haplo-insufficent yeast genes	Deutschbauer, et al. [8]
Over-expression sensitive yeast genes	Makanae, et al. [9], Sopko, et al. [10]
Essential yeast genes	Saccharomyces Genome Deletion Project [4]<br>http://www-sequence.stanford.edu/group/yeast_deletion_project/Essential_ORFs.txt

Supplementary Note 1

This note details results of expression sensitivity / noise intolerance and principal topology analyses for RAP1, which we excluded from the main Result sections due to its outlier landscape topology.

When quantified for expression sensitivity and noise intolerance (analyses presented in Figure 4), RAP1 has 4.2% expression sensitivity and -1.2% noise intolerance (i.e. increasing noise is beneficial), thus breaking the agreement between magnitudes of these two quantities observed across the other assayed genes. When decomposed into principal topologies (analysis presented in Figure 5), RAP1 has loadings of 2 and -2.2 for principal topologies 1and 2, respectively, making it a sole outlier.

The RAP1 landscape shows a monotonous increase of fitness with mean expression, with no signs of saturation at or even above the estimated wild-type mean expression, as well as beneficial fitness effects of increasing expression noise. This is similar to the landscapes observed for ENO2 and RPL3, two genes for which the estimated wild-type mean expression lies far above the mean expression range covered by their fitness landscape (Supplementary Figure 7).

Together this therefore suggests that either wild-type mean expression from the endogenous RAP1 promoter was mis-estimated, or that in the specific experimental selection conditions RAP1 wild-type mean expression is not well aligned with optimal mean expression levels. Thus, the RAP1 results warrant further experimental investigation.

Supplementary References

1 Keren, L. *et al.* Massively Parallel Interrogation of the Effects of Gene Expression Levels on Fitness. *Cell* **166**, 1282-1294.e1218, doi:10.1016/j.cell.2016.07.024 (2016).

2 Sharon, E. *et al.* Probing the effect of promoters on noise in gene expression using thousands of designed sequences. *Genome Research* **24**, 1698-1706, doi:10.1101/gr.168773.113 (2014).

3 Hausser, J., Mayo, A., Keren, L. & Alon, U. Central dogma rates and the trade-off between precision and economy in gene expression. *Nat Commun* **10**, 68, doi:10.1038/s41467-018-07391-8 (2019).

4 Giaever, G. *et al.* Functional profiling of the Saccharomyces cerevisiae genome. *Nature* **418**, 387-391, doi:10.1038/nature00935 (2002).

5 Sharon, E. *et al.* Inferring gene regulatory logic from high-throughput measurements of thousands of systematically designed promoters. *Nature Biotechnology* **30**, 521-530, doi:10.1038/nbt.2205 (2012).

6 Newman, J. R. S. *et al.* Single-cell proteomic analysis of S. cerevisiae reveals the architecture of biological noise. *Nature* **441**, 840-846, doi:10.1038/nature04785 (2006).

7 Stewart-Ornstein, J., Weissman, J. S. & El-Samad, H. Cellular noise regulons underlie fluctuations in Saccharomyces cerevisiae. *Molecular Cell* **45**, 483-493, doi:10.1016/j.molcel.2011.11.035 (2012).

8 Deutschbauer, A. M. *et al.* Mechanisms of haploinsufficiency revealed by genome-wide profiling in yeast. *Genetics* **169**, 1915-1925, doi:10.1534/genetics.104.036871 (2005).

9 Makanae, K., Kintaka, R., Makino, T., Kitano, H. & Moriya, H. Identification of dosage-sensitive genes in Saccharomyces cerevisiae using the genetic tug-of-war method. *Genome Research* **23**, 300-311, doi:10.1101/gr.146662.112 (2013).

10 Sopko, R. *et al.* Mapping pathways and phenotypes by systematic gene overexpression. *Molecular Cell* **21**, 319-330, doi:10.1016/j.molcel.2005.12.011 (2006).
