# Supplementary material for: Empirical mean-noise fitness landscapes reveal the fitness impact of gene expression noise
Source: Nat Commun. 2019 Jul 18;10:3180. doi: 10.1038/s41467-019-11116-w (PMC6639414; doi:10.1038/s41467-019-11116-w)
Supplement: Supplementary file 3 — Reporting Summary [file 41467_2019_11116_MOESM3_ESM.pdf]

nature research

Corresponding author(s): Joern M. Schmiedel, Ben Lehner

Last updated by author(s): Jun 13, 2019

Reporting Summary

Nature Research wishes to improve the reproducibility of the work that we publish. This form provides structure for consistency and transparency in reporting. For further information on Nature Research policies, see Authors & Referees and the Editorial Policy Checklist.

Statistics

For all statistical analyses, confirm that the following items are present in the figure legend, table legend, main text, or Methods section.

n/a	Confirmed	
☐	☒	The exact sample size (*n*) for each experimental group/condition, given as a discrete number and unit of measurement
☒	☐	A statement on whether measurements were taken from distinct samples or whether the same sample was measured repeatedly
☐	☒	The statistical test(s) used AND whether they are one- or two-sided<br>*Only common tests should be described solely by name; describe more complex techniques in the Methods section.*
☐	☒	A description of all covariates tested
☐	☒	A description of any assumptions or corrections, such as tests of normality and adjustment for multiple comparisons
☐	☒	A full description of the statistical parameters including central tendency (e.g. means) or other basic estimates (e.g. regression coefficient) AND variation (e.g. standard deviation) or associated estimates of uncertainty (e.g. confidence intervals)
☒	☐	For null hypothesis testing, the test statistic (e.g. *F*, *t*, *r*) with confidence intervals, effect sizes, degrees of freedom and *P* value noted<br>*Give P values as exact values whenever suitable.*
☒	☐	For Bayesian analysis, information on the choice of priors and Markov chain Monte Carlo settings
☒	☐	For hierarchical and complex designs, identification of the appropriate level for tests and full reporting of outcomes
☐	☒	Estimates of effect sizes (e.g. Cohen's *d*, Pearson's *r*), indicating how they were calculated

Our web collection on statistics for biologists contains articles on many of the points above.

Software and code

Policy information about availability of computer code

Data collection	no software was used to collect data
Data analysis	All analysis was performed using Matlab version R2014b. All code to repeat the analyses can be found at https://github.com/lehner-lab/mean-noise-fitness-landscapes

For manuscripts utilizing custom algorithms or software that are central to the research but not yet described in published literature, software must be made available to editors/reviewers. We strongly encourage code deposition in a community repository (e.g. GitHub). See the Nature Research guidelines for submitting code & software for further information.

Data

Policy information about availability of data

All manuscripts must include a data availability statement. This statement should provide the following information, where applicable:

- Accession codes, unique identifiers, or web links for publicly available datasets
- A list of figures that have associated raw data
- A description of any restrictions on data availability

No primary data have been generated in this study. All data sources are listed in the Data acquisition section. The source data underlying Figs 2d, 4b–d and 5a are provided as a Source Data file. Pre-processed data can also be found at https://github.com/lehner-lab/mean-noise-fitness-landscapes.

Field-specific reporting

Please select the one below that is the best fit for your research. If you are not sure, read the appropriate sections before making your selection.

☒ Life sciences ☐ Behavioural & social sciences ☐ Ecological, evolutionary & environmental sciences

For a reference copy of the document with all sections, see nature.com/documents/nr-reporting-summary-flat.pdf

Life sciences study design

All studies must disclose on these points even when the disclosure is negative.

Sample size	No sample sizes were pre-determined/chosen. All available data was used for the analyses.
Data exclusions	Promoters: Promoters were checked for consistency of mean expression estimates between driving YFP on a plasmid 38 and driving YFP from the HIS3 locus 24. A linear model fit to the log2-transformed mean expression data was used to transform the plasmid-derived data in order to make the two studies comparable (Matlab function polyfit with degree 1, i.e. slope and intercept were fit). 11 of 120 promoters that showed a log2-derivation of more than 0.5 between mean expression estimates in both studies were discarded (Supplementary Fig. 1a). Another 6 promoters that had a median fitness error estimate over all promoter-gene combinations greater than 0.1 were discarded (Supplementary Fig. 1b). Finally, to restrict our analysis to a sufficiently homogenously populated core region in the mean-noise space, 24 promoters with mean expression below 2 or above 6 log2-expression units were discarded (Supplementary Fig. 1c). Because our subsequent analyses are focused on the fitness effects around the wild-type expression of genes, only those 33 of 85 genes that have an estimated mean expression output of their wild-type promoters that lies in the centre of the analysed expression range (between 3 and 5 log2-expression units) were considered (Supplementary Fig. 1d). Additionally, for the transcription factors ABF1, MIG1 and RAP1 several promoter-gene pairs (3, 5 and 2, respectively) were discarded from our analysis because the promoters contain predicted binding motifs for these genes.
Replication	No replication attempts were performed, since this study is based on only one previously published dataset.
Randomization	No samples were allocated into experimental groups.
Blinding	No blinding was performed as there was no group allocation.

Reporting for specific materials, systems and methods

We require information from authors about some types of materials, experimental systems and methods used in many studies. Here, indicate whether each material, system or method listed is relevant to your study. If you are not sure if a list item applies to your research, read the appropriate section before selecting a response.

Materials & experimental systems

n/a	Involved in the study	
☒	☐	Antibodies
☒	☐	Eukaryotic cell lines
☒	☐	Palaeontology
☒	☐	Animals and other organisms
☒	☐	Human research participants
☒	☐	Clinical data

Methods

n/a	Involved in the study	
☒	☐	ChIP-seq
☒	☐	Flow cytometry
☒	☐	MRI-based neuroimaging
